# Exploring the obesity concerns of British Pakistani women living in deprived inner‐city areas: A qualitative study

**DOI:** 10.1111/hex.13527

**Published:** 2022-05-14

**Authors:** Halima Iqbal, Jane West, Rosemary R. C. McEachan, Melanie Haith‐Cooper

**Affiliations:** ^1^ Born in Bradford Bradford Institute for Health Research, Bradford Teaching Hospital NHS Foundation Trust Bradford UK; ^2^ Faculty of Health Studies University of Bradford Bradford UK

**Keywords:** Bradford, British Pakistani women, deprived areas, obesity concerns, obesity research agenda, qualitative, research priority setting

## Abstract

**Introduction:**

British South Asians have a higher prevalence of overweight and obesity than the wider population. Bradford (UK), with its high Pakistani presence and levels of economic deprivation, has exceptionally high instances, especially in deprived areas where many Pakistanis reside. British Pakistani women in Bradford are more likely to be overweight and obese. There is uncertainty on how these women can be aided to manage their weight. Therefore, the objective of this study was to explore the obesity concerns of Pakistani women living in deprived inner‐city areas of Bradford.

**Methods:**

Three focus groups interviews were carried out with 23 Pakistani women living in deprived areas of Bradford. Data were analysed thematically.

**Results:**

This exploratory study identified a wide range of concerns that women had around managing their weight. Participants disclosed distrust in information given around medication, conflicting dietary information and reported low levels of trust in women‐only organized physical activities. Cultural barriers were identified, which included the gender role of the woman, the lack of culturally appropriate dietary advice, cultural misunderstandings of what constitutes a healthy diet and healthy weight, the lack of culturally suitable exercise facilities and conforming to family and community expectations. Other concerns were language barriers around a lack of understanding, the inability to read Urdu and reliance on others to translate information.

**Conclusion:**

These findings have implications for researchers, local authorities, policy makers and others with an interest in reducing the rates of obesity in this population. Recommendations include training health practitioners to be culturally aware of the diet and eating practices of this community, exploring different ways to support socially isolated women to be more physically active at home, addressing physical activity and diet misconceptions and designing obesity management information materials appropriate for a range of literacy levels.

**Patient or Public Contribution:**

Public contributors were involved in the development of the interview guide and design of the research. A pilot focus group with participants not included in the present paper was used to help test and refine the focus group questions. Interview transcripts were member checked by participants, and participants assisted with data analysis.

## 1 INTRODUCTION

Obesity is an increasing global, public health issue. Excess levels of fat in the body increase the risk of disease,^1^ and obesity is a major risk factor for developing a range of conditions including cardiovascular disease, type 2 diabetes, muscular disorders, respiratory conditions and a host of psychological problems.^2^ Recently, obesity has emerged as a key risk factor for hospitalization and death due to Covid‐19 outcomes.^3^, ^4^, ^5^ British South Asians and socioeconomically deprived populations have higher rates of obesity compared to the wider population.^6^ South Asian populations store more excess fat in their abdominal region compared with White European populations, which is associated with elevated disease risk.^7^ South Asian people also develop obesity‐related disease at lower body mass index (BMI) cutoff points than the wider population, prompting the World Health Organization to suggest using lower thresholds to define overweight and obesity in this population.^8^ Individuals with a BMI over 35 have an 80‐fold increased risk of diabetes than those of a normal BMI,^9^ and the prevalence of type 2 diabetes is reported to be strikingly higher in South Asian populations than for other ethnic groups.^10^, ^11^ The northern city of Bradford, in England, UK, has a strong Pakistani presence and high levels of economic deprivation.^12^ Bradford district ranks as the 13th most deprived local authority in England (from a total of 317, with 1 being the most deprived) and the 2nd most deprived in the Yorkshire and Humber region.^13^ The most deprived wards of the district include the highest percentage of Pakistani residents.^14^ There is a high prevalence of overweight and obesity in Bradford district^15^ compared to the national average,^16^ especially in more deprived areas, where rates are double those in more affluent areas of the district.^17^ The Pakistani population in Bradford has significantly high rates of obesity.^18^ Bradford is also home to the largest number of people living with type 2 diabetes in the United Kingdom.^19^ Despite this, British South Asian people are less likely to exercise or follow a healthy diet compared to the general population.^20^ Within the South Asian population, however, some subgroups experience disproportionately higher rates of obesity, such as British Pakistani women.^21^, ^22^ There is a need to focus attention on improving the health of this population and reduce their risk of type 2 diabetes and other obesity‐related health risks.

**Figure 1:**
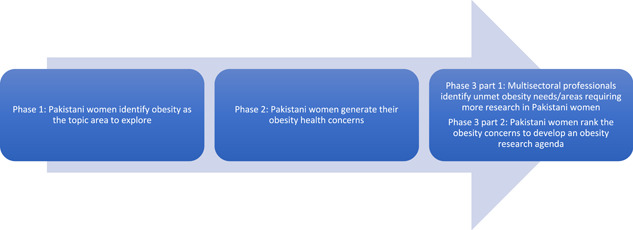
Phases of the research.

This study was followed by a study conducted to ascertain the unmet health needs of Pakistani women living in deprived areas of Bradford, in which issues around diet and physical activity featured strongly as a theme (Phase 1; under review). See Figure 1 for the different phases of this study. The aim of the exploratory qualitative study reported in this article (Phase 2) was to identify the obesity health concerns of British Pakistani women with a view to inform a subsequent research priority‐setting exercise with two distinct phases (Phase 3 part 1 and part 2).^23^ Research priority setting helps to direct the most effective use of resources, such as time and funds, and research capacity, to ensure an optimal health impact.^24^ Setting priorities for research has the potential to reduce health inequalities by producing research that is more effective in overcoming health issues. Involving communities ensures fairness and identifies the needs of vulnerable groups.^25^ Despite the apparent benefits to involving such groups, evidence confirms that marginalized individuals are either seldom heard or left out completely in health priority setting.^26^, ^27^ As a result, research questions that are pertinent to people may be overlooked or undervalued,^28^ further marginalizing vulnerable groups. South Asian populations are underrepresented in health research,^29^ and there is no evidence of research prioritization exercises conducted with any South Asian groups in the area of obesity.^30^ Therefore, the research question that this study aimed to address was follows: What are the obesity concerns of Pakistani women living in deprived inner‐city areas of Bradford?

## 2 METHODS

### 2.1 Design

Given the paucity of studies exploring the obesity concerns of British Pakistani women and the known barriers in engaging seldom heard ethnic groups in research, we used a feminist participatory action research^31^ design to comprehensively explore women's obesity concerns. This approach allows for the identification of women's obesity concerns from their description of lived experience and lessens the researcher impact and imbalance of power inherent in the researcher and participant relationship.^32^ It should be noted that the corresponding author, H. I., shares similar characteristics to the participants in that she is a Muslim, Pakistani woman also living in Bradford. The decision to undertake focus groups was informed by support for this method from public and patient involvement in the project. Before this study, public contributors who reflected the target population reported that Pakistani women may engage more with a focus group with others that shared the same characteristics as them, rather than one‐to‐one interviews. Focus groups are particularly useful in generating data from marginalized groups who may feel anxious about participating in research,^33^ with ethnically homogeneous focus groups appearing to be more at ease discussing ethnic and cultural differences than more heterogeneous ones.^34^ Research prioritization involving South Asian communities have solely utilized the focus group method, noting its effectiveness for eliciting data from this particular population.^35^, ^36^, ^37^ Ethical approval was granted from the University of Bradford (Reference E722).

### 2.2 Sampling and recruitment

We used purposive sampling by location (deprived areas of Bradford), ethnicity (Pakistani) and religion (Islam) to recruit both English‐speaking and non‐English‐speaking adult women (18+ years). Muslim, Pakistani women specifically were included because they have distinct cultural and religious needs that prevent them from living a healthy lifestyle, which is cited as one reason for failed interventions in this group.^38^ Three community and schools settings were targeted in which to run three focus groups, which is the recommended number for data to be collected sufficiently to facilitate the emergence of patterns and themes across the focus groups.^39^ All three settings were located within the most deprived inner‐city wards of Bradford across most domains of deprivation (income, employment, education skills and training, health deprivation and disability, crime, barriers to housing and services and living environment).^40^ Eligible women in two of the focus groups were existing members of scheduled, weekly meetings. One was a parents' group held at a school (*n* = 9) and the other at a community organization (*n* = 9) that actively work with Black and minority ethnic groups. These groups were also specifically targeted because the women had pre‐existing relationships, which are found to be a key factor determining participation in focus groups.^41^ Initial contact was made with gatekeepers, both of whom were group leaders, at both settings who assisted with recruitment of women who fulfil the eligibility criteria. Pre‐existing positive relationships between the gatekeeper and participants can be used by a researcher to facilitate the social access to potential participants.^42^ Information sheets and consent forms were given to all Pakistani women who expressed an interest in participating. For non‐English‐speaking women, the gatekeepers assisted in translating the study aims into Urdu, along with the information sheet and consent forms. The third focus group was held at an Islamic faith school (*n* = 5), wherein nearly all students were of Pakistani heritage. This focus group included women who had no prior relationship, and yet, their children were students at the school and the women regularly attended the monthly school assembly. The deputy head teacher at the school was approached and allowed the main author a 15‐min segment at the end of assembly to discuss the study and recruit women who fit the eligibility criteria. Settings in which the target group normally congregate should be considered by the researcher,^43^ and will increase the likelihood of participation by seldom heard communities.^44^ We aimed to recruit our sample to ensure between four and eight participants, as is recommended.^45^ Women provided informed consent at the start and end of the focus group to ensure that they were still happy for the inclusion of their data.

### 2.3 Data collection

All three focus groups were audio‐recorded and conducted by H. I. in June and July 2019, lasting 1 h to 90 min each. A semistructured interview guide was developed. During its development, two other guides exploring research priorities in focus groups with South Asian communities were examined to determine the most appropriate terminology to use.^36^, ^37^ Public contributors also assisted in the wording of the guide. The guide was pilot‐tested and iteratively refined to make it more comprehendible to women with various literacy levels. The interview guide is presented in Table 1.

**Table 1:** Interview topic guide

1. *What are your concerns around healthy eating? (Probed for: cooking for others, knowledge of portion sizes)* 2. *What are your concerns around physical activity? (Probed for barriers)* 3. *Are there any things making it difficult for you to be a healthy weight? (Probed for weddings, Eid, cultural celebrations)* 4. *Are there any topics around your weight that you think are important to look at?* 5. *What do you think research should look at to help you be a healthy weight?* 6. *What specific improvements would you like to see others make in obesity research* 7. *What could be done to help you manage your weight?* 8. *Are there any other topics around obesity that you think should be looked at in more detail?*

### 2.4 Data analysis

Focus group data were transcribed verbatim, with participant information being anonymized to protect confidentiality. Transcriptions were verified for accuracy by participants who attended the existing group sessions, and to ensure that the key issues from the data were a true reflection of what was discussed during the focus groups. The transcribed data were analysed using inductive thematic analysis, and semantic codes and themes were identified in the data to reduce the risk of making assumptions from the data.^46^ The transcribed data were managed using Nvivo® qualitative software (version 12.0). Themes were discussed and developed between H. I. and M. C. during group meetings. Member checking with participants from two of the focus groups (*n* = 15) was done to further enhance internal validity.^47^

## 3 FINDINGS

In total, 23 women participated across three focus groups. The women's ages ranged from 18 to 65 years. Seven women did not speak English. H. I. is proficient in Urdu. Gatekeepers and other participants also assisted in translation where needed. Most participants had children (*n* = 19) and were married (*n* = 19) and unmarried (*n* = 4). Each group included participants of mixed ages. The demographic details of the participants are shown in Table 2. Analysis revealed three main themes concerning Pakistani women's obesity‐related concerns: (1) distrust, (2) cultural barriers and (3) language barriers. These themes and corresponding codes are shown in Table 3.

**Table 2:** Demographic information of the participants (*n* = 23)

	Setting	Pseudonym	Age	English speaking?	Married?	Children?
Focus group 1 (*n* = 5)	Faith school	Ayesha	50	✓	✓	✓
		Sobia	32	✓	✓	✓
		Naseem	28	✓	✓	✓
		Zainab	38	✓	✓	✓
		Ruksana	36	✓	✓	✓
Focus group 2 (*n* = 9)	Primary school	Zara	27	✓	✓	✓
		Shamim	35	X	✓	✓
		Sanna	32	✓	✓	✓
		Salma	42	✓	✓	✓
		Shaheen	37	✓	✓	✓
		Sugra	40	✓	✓	✓
		Iram	32	X	✓	✓
		Kausar	34	X	✓	✓
		Farzana	36	X	✓	✓
Focus group 3 (*n* = 9)	Community organization	Shazia	65	✓	✓	✓
		Taiba	56	X	✓	✓
		Sadia	26	✓	X	X
		Maryam	43	X	✓	✓
		Fathima	20	✓	X	X
		Hannah	33	✓	X	X
		Rashida	58	✓	✓	✓
		Nusret	48	X	✓	✓
		Saba	18	✓	X	X

**Table 3:** Themes and codes

Theme 1: Distrust	Theme 2: Cultural barriers	Theme 3: Language barriers
Code:	Code:	Code:
− Information given around medication. − Conflicting dietary information given by health professionals. − Women‐only organized physical activities.	− Gender role of the woman. − Lack of culturally appropriate dietary advice. − Lack of understanding of healthy/unhealthy diet. − Lack of culturally appropriate exercise facilities around physical activity. − Conforming to family and community expectations. − Lack of understanding of healthy weight.	− Lack of understanding. − Inability to read Urdu. − Reluctance to attend weight management services. − Reliance on others for information.

### 3.1 Distrust

Many participants expressed a lack of trust in authority and felt that the distrust they felt persisted as a barrier to managing their weight. Almost half of the participants (*n*  = 10) identified that they were living with obesity‐related health conditions, namely, type 2 diabetes, high blood pressure, hypertension and stroke. They expressed frustration at the lack of information given to them by healthcare professionals when prescribing them medications, and as a result, had mistrust in their advice. Participants felt that these medicines made them gain more weight and that they were not informed of this at the time:

*Drs don't tell you that these are the side effects of these medications. Generally, any medication ending with ‘ine’ like olanzapine, mirtazapine, they put weight on, but they don't tell you that*. (Shazia)

Participants expressed concern at being unsure of what dietary advice to follow to lose weight because advice from professionals was inconsistent. This lack of consistency led to women lacking trust in the advice that they were given by professionals. This lack of trust led to women not following the advice given to them by professionals, which meant that they did not lose weight. Participants disclosed that professionals did not know enough about Pakistani culture and that this could be a reason why advice was inconsistent:

*Like, she's diabetic and may be told to have less bread and another diabetic person might be told to have as much bread as they want but have less roti. I doubt they even know what roti is. I don't trust what they say to be fair*. (Zara)

Participants reported that they did not trust the ladies‐only fitness facilities that were available to them to support weight loss. This was a barrier in accessing exercise for these women. Examples given by participants included ladies‐only swimming classes in which male lifeguards would enter the swimming area, which participants stated they were not made aware of before booking the sessions. Distrust in exercising in ladies' sections of gyms was most frequently cited as a concern for participants:

*Like, they have women's only gyms and stuff near me but we (referring to herself and her sister) won't go because men walk in whenever they want even though it's meant to be a women's only space, and they got cameras in there that the male staff can see in their office so no way do I trust it*. (Ayesha)

### 3.2 Cultural barriers

Participants explained that the cultural norm for them as Pakistani women was to be responsible for domestic labour. This proved to be a barrier in engaging in behaviours that would help them manage their weight. It was reported that the men of the house worked unskilled, low‐paid jobs such as taxi drivers and in take‐away outlets during unsocial hours, which meant that women were very busy running household chores, which made it exceptionally tedious for the participant, who then had little energy to exercise or prepare healthy foods, despite desiring to do so.

*I would love to cook healthy food for my family but when you have to drop off and pick up the kids from school, and then mosque, and do all the errands outside, I can't be expected to make something healthy on top or exercise*. (Zainab)

Many participants expressed concerns for relatives and neighbours who did not leave the house due to cultural expectations for women to remain indoors, by either their own family (parents and siblings), their husband or their in‐laws, leading to a barrier for women in engaging in outdoor physical activity:

*They have families and in‐laws they're living with, and they won't let them out or their husbands won't. How are they meant to have a healthy life then?* (Salma)

Most participants felt that a barrier to engaging in physical activity was the lack of culturally suitable exercise facilities available to them. Music was often listed as a barrier in engaging in exercise classes as music is forbidden in Islam. Women expressed that they felt guilty exercising with background music, and some had walked out of the class part way through for this reason.

*I have been to exercise classes before, but they had music on, so I felt bad I left in the middle of it didn't go back again. You know music is haram (forbidden) in Islam*. (Fathima)

The most dissatisfaction was shown at the lack of ladies‐only exercise facilities:

*Considering that this is Bradford and how many Muslims and Pakistanis there are here, there aren't enough things for women, and we seem to be the ones that need it the most*. (Ruksana)

Participants discussed that they would not leave the house for the purpose of engaging in physical activity in cold weather due to the perception that it would cause illness. Participants disclosed that women who had moved to the United Kingdom from Pakistan in particular, and older Pakistani women, would not attend outdoor swimming sessions due to fears of catching pneumonia:

*That's why they won't go swimming, even if it's ladies only. They think they'll get pneumonia from cold water*. (Sadia)

Participants discussed concerns around the lack of culturally appropriate dietary advice. They identified an awareness of government recommendations for which foods should be consumed and their quantities, but were unaware of how this translated into culturally appropriate foods that they consumed at home with their families that originated from the Indian subcontinent. Foods mentioned included halwa, pilau, roti salan, mithai and panjiri, all highly calorific foods when cooked in the traditional way.

*Have a chapatti? Like, how would you even say it? Its ok for the English, you can say this portion of that food but how for Asian?* (Sanna)

Some participants mentioned that traditional food constituted the bulk of their diet and that these foods lost their authenticity and taste if cooked in a way deemed healthier. Despite this, participants desired education on cooking palatable traditional foods in a healthier way.

*It would be good if we were taught how to cook Asian food in a way that's healthy but still tastes good. I think that's a big problem with us Pakistanis*. (Naseem)

Many participants mentioned the pressure to conform to family and societal expectations in the Pakistani community as a barrier to managing their weight. For instance, they felt pressured to mirror unhealthy eating behaviours in a variety of settings in day‐to‐day life and did so to avoid disagreements in the house. They mentioned that their husbands and children requested highly calorific foods and thus, they felt pressured to cook it. On occasion, they cooked their own healthier meals separately, but it was too time consuming, so they reverted to cooking solely for the family. When they did try eating healthier, family members complained and would pressure them to turn back to their previous cooking habits:

*(translated) I decided I was going to eat healthy last Ramadan so didn't make samosas. My mother‐in‐law found out and said I was selfish and that it wasn't fair on the kids or her son, and I felt really bad, so I made them for the rest of rozay (fasting in Ramadan)*. (Farzana)

A further example was given of the expectation to eat unhealthy foods at cultural and religious events. Foods listed were samosas, pakoras, fried rice and Asian sweets and desserts. Participants discussed that these events occurred often, and especially during Ramadan when they broke their fasts at the houses of relatives and friends. Asian weddings and celebratory events leading up to the wedding date were filled with these high‐fat, low‐nutrient foods that were expected to be consumed:

*People don't let you eat healthy if you go to their house or like, on Eid. They get offended if you don't eat whatever they've put out for you and its always fried food and desserts like mithai*. (Sugra)

Participants expressed concerns about the cultural beliefs surrounding what a healthy diet looked like, especially for Pakistani women. The overconsumption of high‐sugar and high‐fat cultural foods in the Pakistani diet was frequently mentioned, with a cultural belief that these foods were perceived to be healthy or would ward off poor health. For instance, one participant remarked:

*From Autumn through to Winter, lots of women eat panjiri (a high fat dessert made from ghee, sugar and nuts). It stops you from getting ill if you eat a little bowl of it two or three times a day, but they don't realise they're putting on loads of weight*. (Salma)

Many participants explained that one pressing concern pertaining to obesity was the widespread misunderstanding of what constituted a healthy weight due to cultural beliefs. There was consensus across the groups that excess weight was considered healthy and slimness was frowned upon by many Pakistanis, despite the participants themselves acknowledging that excess weight was not ideal. They expressed that a lot of the misconceptions originated from Pakistan and still prevail. Participants discussed that these beliefs were imposed on children from a young age. Participants who were a healthy weight spoke of how they were constantly pressured to gain weight because their slender frames were considered unhealthy:

(translated) they don't let you live. They always say to me I'm so skinny and should put weight on but I'm healthy. Everybody else is fat but in our culture, its normal to be fat so I'm not normal. (Shamim)

### 3.3 Language barriers

Both English‐speaking and non‐English‐speaking participants expressed concerns that it was difficult for people who did not speak English to access weight management information such as dietary advice or advice on the benefits of physical activity. Non‐English‐speaking women specifically expressed that they were unable to understand weight management information as it was provided in English and was not always available in their spoken language:

(translated) *I don't understand what they're saying because I can't speak English. I just smile and nod*. (Taiba)

Furthermore, some participants stated that even when weight management information was written in the Urdu language, they were unable to read it because they did not speak or read Urdu, and their spoken language, Mirpuri, has no written form:

(translated) *They have the information written in Urdu, but I can't read Urdu. I only speak Mirpuri*. (Kausar)

Language barriers often meant that Pakistani women lacked essential knowledge on how to live a healthy lifestyle, so relied on their wider social networks to obtain weight management advice, which, they did not always agree with made sense, and yet took on board regardless:

*(translated) I want to lose weight but can't speak English so didn't speak to anyone (a health professional) about it. My neighbour said to eat a bowl of nuts when I got hungry. I thought nuts made people fatter, so I don't know. I'm giving it a try. I started it two days ago*. (Farzana)

Participants mentioned that an obesity concern was not being able to attend exercise classes in their local area or at the gym because the instructor was speaking in English, and they could not understand what was being said. Participants further discussed that they, as non‐English speakers, or other non‐English‐speaking Pakistani women they knew had been referred by their general practitioner to attend diabetes education courses, and yet, they were either reluctant to attend altogether, or attended one session only, because they felt that they would not derive benefit from the sessions due to their inability to understand English:

*(translated) I went to one of those diabetes groups and they were talking about food, but I didn't know what they meant or what the foods were that they talked about. I didn't go again after that*. (Taiba)

Participants disclosed that they assisted non‐English‐speaking relatives in acquiring weight management information because without doing so, they were concerned that they would lack the necessary information required to manage their weight:

I know people who don't speak English have a problem with it here in Bradford. Like, I had to call someone for my mother‐in‐law because she didn't know what she should or shouldn't be doing when it came to her diabetes. (Shaheen)

## 4 DISCUSSION

### 4.1 Summary of key findings

We undertook this study to identify the obesity health concerns of Pakistani women living in deprived inner‐city areas of Bradford. To our knowledge, this is the first study to identify and present the obesity concerns of British Pakistani women living in deprived areas. Women expressed distrust as a concern, which included the lack of trust in information provided about medication by professionals, mistrust in contradictory information around diet and a lack of trust in women‐only organized physical activity. The theme of cultural barriers revealed that the obesity concerns of Pakistani women centred around the gender role of the Pakistani woman, the lack of culturally appropriate dietary advice, the lack of culturally suitable exercise facilities and pressure to conform to family and community expectations. For non‐English‐speaking participants, obesity concerns were the prevalence of language barriers, which meant that they were unable to understand weight management advice, the inability to read weight management advice written in Urdu, having to rely on their social networks for dietary advice and the lack of language‐appropriate weight management services.

### 4.2 Comparison to other research, and implications for practice and research

These findings confirm and build upon prior research. Like our study, access to health promotion has been found to be hindered by the failure of health providers to offer practical diet advice that is culturally inappropriate.^48^ It is noted elsewhere that access to a linguistically and culturally familiar dietician is important to South Asian women.^49^, ^50^ Similar to our study, language and cultural needs of Black and minority ethnic communities act as major barriers for dieticians to provide care for these communities.^51^ However, no previous research could be found around professionals relaying contradictory dietary advice. Research has examined contradictory messages around nutrition; yet, no research was identified in the context of conflicting dietary advice delivered by health professionals, but in terms of mixed messages in the media.^52^ The women in the current study stated that they felt that the contradictory diet advice given by professionals was due to the lack of cultural awareness of foods consumed in the Pakistani diet among these professionals. It is thus important that health practitioners such as GPs and dieticians are trained to be aware of cultural customs and traditions surrounding the food and eating practices of this population so that culturally appropriate advice can be given, which can provide value for this community.

Pakistani women in this study reported that they do not have time to engage in health‐promoting activities due to their gender role in the family. Family life is considered essential and sacred within Islam, and women have been assigned the role of taking care of the family.^22^ As such, the interests and needs of the family are put first and exercise beyond housework is considered selfish by some South Asian women^22^ as well as by their families and communities.^53^, ^54^ We found that gender norms impeded opportunities for Pakistani women to engage in physical activity. Our findings support other studies examining the health behaviours of Pakistani women in relation to gender norms in the family.^53^, ^54^, ^55^ An example of one such barrier found in both our study and the wider literature is the cultural expectation for women to remain indoors, resulting in infrequent outdoor physical activity.^53^, ^56^, ^57^ Future support could focus on helping women who do not leave their homes. One way to do this could be to educate them on how they can be more active doing home‐based activities as is outlined by the NHS and Public Health England advice. An example of this is the 2020 Active at Home booklet launched by Public Health England and Sport England for older adults,^58^ which may prove very useful in increasing physical activity levels in Pakistani women. In addition to promoting exercise at home for these women, wider weight management services need to consider women's home and local contexts. The development of services delivered at the grassroots level may be more impactful than weight management programmes currently being promoted through hospitals or other appointments that women are unlikely to attend due to the reasons identified in this study. These grassroots services could be home‐based services or services that women could attend within frequently visited settings such as faith settings, schools, local community groups and local pharmacies. Faith settings may be particularly useful as a delivery channel for obesity interventions^59^ as evidence suggests that health promotion interventions in these settings have a high reach.^60^

Women in the present study indicated that they lacked knowledge of commonly consumed Asian foods that were high in fat and sugar. This is in line with other research that has found mixed knowledge in South Asians of high fat and high sugar contents of traditional South Asian foods.^61^, ^62^ Similarly, women in the present study felt that consumption of traditional high‐fat and sugary foods was important for good health and should be regularly consumed. There is a paucity of literature on the misconceptions of this population on the benefit of these foods to their health. In addition to the lack of studies on dietary misconceptions, misconceptions surrounding physical activity in this population have also not been widely explored in the literature. It is recommended that not only should education efforts be targeted towards addressing misconceptions surrounding physical activity and diet in this population but also that educational interventions for this group are culturally appropriate to ensure that they have a greater impact. Examples of good practice can be found in the literature, in which educational interventions targeting health promotion in South Asians resulted in positive learning, highlighting the success of the intervention.^63^ Efforts included delivering education in settings that were culturally appropriate to the attendees such as community centres, mosques and schools. In addition, embedding health ambassadors in schools, and recruiting local professionals as education champions proved effective.^63^ It is crucial to note, however, that South Asians are not a homogeneous group.^64^ Pakistani women living in deprived areas of Bradford may differ from other South Asian subpopulations. As such, interventions to address obesity in Pakistani women may need to be specifically tailored for better results.

This study identified that language and literacy problems were an issue for some women, and research has identified that health promotion materials are not always written appropriately for those with low health literacy.^65^ Most individuals with low health literacy are socially deprived, have a limiting health condition or disability, lower levels of education and are from Black and minority ethnic communities.^66^ Researchers and policy makers must consider literacy levels when designing obesity management information materials for Pakistani women living in deprived areas, so that those receiving the education find them appropriate to their language skills.

### 4.3 Study strengths and limitations

This study has several strengths. The women ranged in age and marital status, some were mothers whereas others did not have children. There were differences in their proficiency of spoken English. The sample included both English‐speaking and non‐English‐speaking women, whereas often, non‐English speakers are omitted from research due to issues surrounding language barriers.^67^ Such diversity in the sample meant that a range of different perspectives were captured based on women's experiences, which increased the representativeness of this study and enriched the data. A further strength of this study lies in its reliability. The reliability of the findings was increased in a range of ways in this study. Gatekeepers translated certain words or phrases that H. I. was unaware of, despite her ability to speak Mirpuri and Urdu. Other participants also assisted in interpretation. In this respect, using existing groups in this study proved very beneficial as it was considerably easier to reconvene with these groups. The researcher returned to the participants in two of the focus groups on two separate occasions for verification: first, to verify the transcripts, and then again, to verify the relationship between the themes and codes. By doing so, the risk of misinterpreting the findings was minimized, thus further increasing the reliability of the findings.^68^

This study is not without its limitations. It could be argued that selection bias may have featured in the recruitment of the sample as it is typically highly engaged individuals who engage in research, thus making them unrepresentative of the population.^69^ Despite this, similar findings were reported across all focus groups, adding to the transferability of this study to Pakistani women living in deprived inner‐city areas of Bradford. A further possible limitation of this study could be the risk of bias as a result of the insider status of H. I., who shared similar characteristics to participants. It is plausible that H. I. could have led the discussion based on her pre‐existing knowledge of the population under study. However, the risk of this was minimized by H. I. being extra vigilant about her line of questioning and allowing the conversations to flow naturally with her limited input. This may have thus increased the credibility of this study. Lastly, it is possible that women may have omitted details under the assumption that H. I. may have insider knowledge. However, this risk was minimized by probing and asking for clarity and explicitness when women were being vague. It must also be noted that the findings from this study may not be generalizable to other Pakistani communities elsewhere or other South Asian communities more generally.

## 5 CONCLUSION

The obesity concerns of British Pakistani women living in deprived inner‐city areas of Bradford are strongly linked to their culture and external influences out of their direct control, making it difficult for women to manage their weight. The data from this study can be used by researchers, health practitioners, local authority and policy makers to develop further research and interventions to improve the obesity health outcomes of this population. Recommendations include training health practitioners to be culturally aware of the diet and eating practices of this community, exploring ways to support women who are socially isolated on how to be more physically active at home, addressing misconceptions surrounding physical activity and diet in this population and designing obesity management information materials appropriate for a range of literacy levels.

## AUTHOR CONTRIBUTIONS

Halima Iqbal conceived the original idea and designed the study. Halima Iqbal managed the project and collected data. The initial coding was done by Halima Iqbal and Melanie Cooper. All the authors were involved in the final analyses and discussions. Halima Iqbal drafted the manuscript, and all authors were involved in revising the manuscript and have given final approval of the version to be published.

## CONFLICTS OF INTEREST

The authors declare no conflicts of interest.

## ETHICS STATEMENT

Ethical approval was granted from the University of Bradford Ethics Committee (Ref No. E722).

## Data Availability

The data that support the findings of this study are available from the corresponding author upon reasonable request.
